# A long-read and short-read transcriptomics approach provides the first high-quality reference transcriptome and genome annotation for *Pseudotsuga menziesii* (Douglas-fir)

**DOI:** 10.1093/g3journal/jkac304

**Published:** 2022-12-01

**Authors:** Vera Marjorie Elauria Velasco, Alyssa Ferreira, Sumaira Zaman, Devin Noordermeer, Ingo Ensminger, Jill L Wegrzyn

**Affiliations:** Department of Biology, University of Toronto, Mississauga, ON L5L 1C8, Canada; Department of Evolution and Ecology, University of Connecticut, Storrs, CT 06269, USA; Department of Evolution and Ecology, University of Connecticut, Storrs, CT 06269, USA; Department of Biology, University of Toronto, Mississauga, ON L5L 1C8, Canada; Graduate Department of Cell and Systems Biology, University of Toronto, Toronto, ON M5S, Canada; Department of Biology, University of Toronto, Mississauga, ON L5L 1C8, Canada; Graduate Department of Cell and Systems Biology, University of Toronto, Toronto, ON M5S, Canada; Graduate Department of Ecology and Evolutionary Biology, University of Toronto, Toronto, ON M5S, Canada; Department of Evolution and Ecology, University of Connecticut, Storrs, CT 06269, USA

**Keywords:** coastal Douglas-fir, *de novo* assembly, full-length isoform, functional annotation, genome annotation, interior Douglas-fir, long noncoding RNA, NovaSeq, PacBio Iso-Seq, *Pseudotsuga menziesii* var. *glauca*, *Pseudotsuga menziesii* var. *menziesii*, reference transcriptome, transcription factors

## Abstract

Douglas-fir (*Pseudotsuga menziesii*) is native to western North America. It grows in a wide range of environmental conditions and is an important timber tree. Although there are several studies on the gene expression responses of Douglas-fir to abiotic cues, the absence of high-quality transcriptome and genome data is a barrier to further investigation. Like for most conifers, the available transcriptome and genome reference dataset for Douglas-fir remains fragmented and requires refinement. We aimed to generate a highly accurate, and complete reference transcriptome and genome annotation. We deep-sequenced the transcriptome of Douglas-fir needles from seedlings that were grown under nonstress control conditions or a combination of heat and drought stress conditions using long-read (LR) and short-read (SR) sequencing platforms. We used 2 computational approaches, namely *de novo* and genome-guided LR transcriptome assembly. Using the LR *de novo* assembly, we identified 1.3X more high-quality transcripts, 1.85X more “complete” genes, and 2.7X more functionally annotated genes compared to the genome-guided assembly approach. We predicted 666 long noncoding RNAs and 12,778 unique protein-coding transcripts including 2,016 putative transcription factors. We leveraged the LR *de novo* assembled transcriptome with paired-end SR and a published single-end SR transcriptome to generate an improved genome annotation. This was conducted with BRAKER2 and refined based on functional annotation, repetitive content, and transcriptome alignment. This high-quality genome annotation has 51,419 unique gene models derived from 322,631 initial predictions. Overall, our informatics approach provides a new reference Douglas-fir transcriptome assembly and genome annotation with considerably improved completeness and functional annotation.

**Significance**

In times of expeditious climate change, high-quality genomic resources are needed for keystone tree species to maintain a healthy forest ecosystem. Here, we present a high-quality transcriptome and improved genome annotation for Douglas-fir, an ecologically and economically important conifer in western North America.

## Introduction

Douglas-fir (*Pseudotsuga menziesii*) is a conifer that exhibits high levels of variation for traits including resistance to heat (Jansen *et al*. 2014) and tolerance to drought (Junker *et al*. 2017). Published Douglas-fir transcriptome showing variability in molecular mechanisms responsive to the environment was conducted using short-read (SR) sequencing technologies. Müller *et al*. (2012) assembled the first Douglas-fir transcriptome *de novo* using 3.6 million reads with an average length of 352 bp. Howe *et al*. (2013), Hess *et al*. (2016), and Cronn *et al*. (2017) identified more than 170,000 unique sequences with only 20% functionally annotated in Douglas-fir transcriptome using SR sequencing.

The only Douglas-fir genome available was also assembled from short fragments (250–635 bp) and long-range linking libraries (3.3–24.8 kbp) obtained from Illumina HiSeq 2500 (Neale *et al*. 2017). It has long scaffolds (N50 340.7 kbp) and long contigs (N50 44 kbp), is highly repetitive, and is estimated that 50% or less is covered with unique *kmers* (*k* = 32). Most of the gene space in the Douglas-fir genome is intronic rather than exonic. The available Douglas-fir genome annotation has a total of 54,830 gene models, of which 83% were functionally annotated. The set was estimated 29% complete via BUSCO's embryophyta lineage.

The pioneering genome and transcriptomes were important in understanding biological variation among and within Douglas-fir varieties (Casola and Koralewski 2018; Howe *et al*. 2020). However, using highly fragmented assemblies as a reference means working with incomplete gene sets and structural annotations. The shortcomings resulting from SR sequencing can be mitigated by long-read (LR) sequencing technologies like Pacific Biosciences Single-Molecule Real-Time isoform sequencing (Iso-Seq; Weirather *et al*. 2017; Byrne *et al*., 2019). Iso-Seq can identify full-length (FL) or nearly FL transcripts at >99.999% consensus read accuracy (Rhoads and Au 2015; Bayega *et al*. 2018) therefore reducing the need for computational assembly (Wu 2016; Kuang *et al*. 2019). Computation approaches include using SRs to correct LR in *Gingko* (Ye *et al*. 2019), sugar pine (*Pinus lambertiana*; Gonzalez-Ibeas *et al*. 2016), and wild cotton (*Gossypium australe*; Feng *et al*. 2019). Well-represented reference genomes were used as a guide for LR transcriptome assembly for grapes (*Vitis vinifera*; Minio *et al*., 2019) and Panicoid grasses (Carvalho *et al*. 2020). LR transcriptome for less-studied species like Japanese Yew (*Taxus cuspidata*; Kuang *et al*. 2019) and *Cattleya*; Li *et al*. 2020) were fully *de novo*. Regardless of approach, LR transcriptome enabled a better understanding of molecular mechanisms in non-model species. Examples are pigment development in *Cattleya* (Li *et al*. 2020) and grapes (Minio *et al*. 2019), and the evolution of photosynthesis in grass (Carvalho *et al*. 2020).

Douglas-fir is lacking high-quality transcriptome and genome annotation resources. Important molecules like transcription factors (TF) and transcription/post-transcription regulators long noncoding RNA (lncRNA; Dykes and Emanueli 2017) in Douglas-fir remain poorly understood (Nystedt *et al*. 2013; Budak *et al*. 2020). TFs in conifers are particularly interesting since many families expanded after the gymnosperm-angiosperm split (Bedon *et al*. 2010; Gramzow *et al*. 2014). So, we used Iso-Seq to sequence transcripts from needles of healthy and stressed Douglas-fir to create a high-confidence transcriptome atlas and compare LR transcriptome assembly with and without the reference genome. We leveraged the LR and SR transcriptome with published assemblies to improve the Douglas-fir genome annotation. Here, we demonstrate the feasibility of generating high-quality transcriptome and genome annotation for Douglas-fir and the utility of this approach for other complex plant genomes.

## Methods

### Plant material

Seeds collected from wild stands of *Pseudotsuga menziesii* var. *menziesii* (Mirb.) Franco (coastal Douglas-fir) and *Pseudotsuga menziesii* var. *glauca* (Mayr) Franco (interior Douglas-fir) were provided by Seed Centre, B.C., Canada. Seeds were soaked in distilled water for 24 h at room temperature, surface-sterilized for 5 h in 30 mL of 3% (w/v) hydrogen peroxide, and stratified in the dark at 4°C for 3 weeks.

Potting mix with final pH of 4.5 and containing 21.6% (v/v) silica sand (Cat. No. 1240s, Bell & Mackenzie, Hamilton, ON, Canada), 13.5% (v/v) sphagnum peat moss (Premier Tech, Rivière-du-Loup, PQ, Canada), 10.8% (v/v) Turface (PROFILE, Buffalo Grove, IL, USA), 7.6% (v/v) coarse perlite (Therm-O-Rock, New Eagle, PA, USA), 3.2% (v/v) medium vermiculite (Therm-O-Rock), 0.1% (v/v) dolomitic limestone (National Lime & Stone, Findlay, OH, USA), and 43.2% (v/v) distilled water was freshly prepared. Seeds were sown on potting mix lightly packed in 168-mL cones and covered with 5 mm silica sand. Seeds were allowed to germinate in a greenhouse for 4 weeks under a maximum of 25°C at midday and a minimum of 17°C at midnight, 17-h photoperiod with at least 400 μmol photons m^−2^ s^−1^ and relative humidity (RH) of 55%. At 4 weeks after planting (wap), seedlings were transferred to 25-L square pots and grown for 6 months under 18-h photoperiod, 400–1,200 μmol photons m^−2^ s^−1^, and 6–36°C simulating 1961–1990 normal B.C. environmental temperatures based on Wang *et al*. (2006) and RH set to 55%. Starting 4 wap, plants were watered once weekly and irrigated twice weekly with fertilizer solutions as prescribed by Wenny and Dumroese (1992) for the initial and accelerated growth phase. Seedlings were acclimated to simulated winter conditions for 2 months in controlled climate chambers set to 8°C/4°C midday/midnight, 8-h photoperiod, and 50–300 μmol photons m^−2^ s^−1^ before another 6 months of growing season in the greenhouse began. By the end of the second growing season, seedlings were acclimated to 22°C midday/14°C midnight and 16-h photoperiod with a minimum of 400 μmol photons m^−2^ s^−1^ in the greenhouse for 6 weeks. Two seedlings remained growing under simulated summer conditions (control). Two seedlings were shifted to growth conditions with increased temperature ranging from 40°C/33°C day/night and water stress by withholding watering (stressed) for another 4 weeks. One-year-old needles from 2 stressed interior Douglas-fir, one control interior Douglas-fir, and one control coastal Douglas-fir were collected and immediately flash-frozen in liquid nitrogen. The needle tissue samples of the 4 Douglas-fir seedlings were then stored at −80°C for later RNA extraction.

### RNA extraction and sequencing

Total RNA was isolated from 200 mg frozen needles using cetyltrimethyl ammonium bromide (CTAB)-based RNA extraction protocol (Chang *et al*. 1993). RNA purification was performed using RNeasy Mini Kit with on-column DNase digestion following the manufacturer's instructions (Qiagen, Germany). RNA was quantified with a Qubit 3.0 fluorometer using the RNA broad-range kit (Life Technologies, Carlsbad, CA, USA). RNA integrity was assessed using an RNA Nano 6000 chip run on an Agilent 2100 Bioanalyzer (Agilent Technologies, Santa Clara, CA, USA) instrument. Control and stressed samples with RNA integrity numbers (RIN) above 8.0 and below 5.0, respectively, were used for FL first-strand cDNA synthesis. PacBio Iso-Seq library was prepared using Smarter Stranded RNA-Seq for Iso-Seq and Pacbio SMARTbell Express Template Prep Kit 2.0 without sheering and size selection. Sequencing was done using one SMRTCell per library, Sequel Chemistry 3.0, and 10-h movie time in PacBio Sequel. Illumina library was prepared from the same cDNA using NEB mRNA stranded library preparation, followed by sequencing with NovaSeq 6000 system using NovaSeq 6000 S4 reagent kit (Illumina, CA, USA) and 1/24 lane per sample to generate 100 bp paired-end reads. cDNA synthesis, library preparation, and sequencing were done at Genome Quebec (Montreal, QC, Canada).

### Iso-Seq LR data quality control and transcriptome assembly

Preprocessing, Iso-Seq quality control, and *de novo* transcriptome assembly were performed using Bioconda Iso-Seq3 version 3.1 following instructions available at https://github.com/PacificBiosciences/IsoSeq_SA3nUP. Iso-Seq subreads with at least one FL sequence were processed to generate circular consensus sequences (CCS) using *ccs*. Barcodes were demultiplexed, 5′ and 3′ cDNA primers were removed from CCS reads, and reads shorter than 50 bp were omitted from the library using *lima* generating FL reads also referred to as “filtered reads.” Poly-A tails and concatemers were removed and multiple SMRTCells were merged using *isoseq3 refine* producing FL nonchimeric (FLNC) reads. Clustering and iterative cluster merging were done with FLNC reads using *isoseq3 cluster* generating unpolished transcripts. Polishing to improve consensus accuracy using *isoseq3 polish* followed by producing “high-quality transcripts.” Following quality control, coding sequences (CDS) from high-quality transcripts were identified using TransDecoder v. 5.3.0 (https://github.com/TransDecoder/TransDecoder). All CDS in each high-quality Iso-Seq library were collapsed at 95% sequence identity using VSEARCH v. 2.4.3 (Rognes *et al*. 2016) to generate a nonredundant set of transcripts or “unique transcripts.” Unique CDS transcripts from all 4 libraries were clustered at 80% sequence identity to generate the LR *de novo* transcriptome. Workflow (Supplementary Fig. 1a) is available in Plant Genomics Lab's Gitlab at https://gitlab.com/PlantGenomicsLab/HQ_Douglas-fir_transcriptome_genome_annotation.

Unique transcripts were also mapped to the Douglas-fir genome (Neale *et al*. 2017) using Gmap v. 2019-06-10 (Wu and Watanabe 2005; Wu *et al*. 2016) using the following parameters: -K 1000000, -L 10000000 –cross-species, –fulllength, –min-trimmed-coverage=.95, –min-identity=.92, and -n 1. gFACs (Caballero and Wegrzyn 2019) was used to create fasta files without introns from Gmap gff3 output. All 4 fasta libraries were clustered at 80% sequence identity using VSEARCH generating the reference genome-guided transcriptome assembly. Workflow (Supplementary Fig. 1a) is available in Plant Genomics Lab's Gitlab.

The quality of transcriptome assemblies was assessed using rnaQUAST (Bushmanova *et al*. 2016). Transcriptome completeness was determined using the Viridiplantae and Eukaryote lineage dataset based on OrthoDB release 10 in BUSCO v. 4 (Simão *et al*. 2015; Waterhouse *et al*. 2018). Functional gene annotation was performed using EnTAP (Hart *et al*. 2019) and Araport11 database (Cheng *et al*. 2016).

### Identification of TF from Iso-Seq LR data

TFs were determined from *de novo* assembled LR transcriptome. TF structural superclass and TF DNA-binding domains were predicted using TFPredict (Eichner *et al*. 2013). GO mapping and annotation using BLAST2GO (Conesa *et al*. 2005) followed. Workflow (Supplementary Fig. 1b) is made available in Plant Genomics Lab's Gitlab.

### Identification of lncRNA from Iso-Seq LR data

LncRNAs in Douglas-fir were predicted from nonredundant Iso-Seq LR transcripts using CREMA (Simopoulos *et al*. 2018). Sequences of Douglas-fir lncRNAs were blasted against *Arabidopsis* lncRNA database (The RNAcentral Consortium *et al*. 2019). BLASTN e-value cutoff was set to 1E − 5, max target =1, and max hsps=1. Workflow (Supplementary Fig. 1c) for prediction and annotation is available in Plant Genomics Lab's Gitlab.

### Illumina SR quality control and transcriptome assembly

Paired SR datasets generated from NovaSeq 6000 were processed and assembled following the workflow (Supplementary Fig. 1d) available in Plant Genomics Lab's Gitlab. Adapter sequences, low-quality reads, and reads with lengths less than 30 bp were removed using Trimmomatic v. 0.36 (Bolger *et al*. 2014). Quality assessment followed using FastQC v. 0.11.7 (https://github.com/s-andrews/FastQC) and MutliQC v. 1.7 (Ewels *et al*. 2016). *De novo* assembly of quality reads into contigs with a minimum length of 350 bp was performed using Trinity v. 2.6.6 (Haas *et al*. 2013). CDS were identified from assembled reads using TransDecoder v. 5.3.0 (https://github.com/TransDecoder/TransDecoder) and then clustered at 95% sequence identity using VSEARCH v.2.4.3 (Rognes *et al*. 2016) to generate a set with only unique transcripts. SR libraries were also clustered at 80% sequence identity to create an SR transcriptome assembly. The quality of transcriptome assembly was assessed using rnaQUAST (Bushmanova *et al*. 2016) and BUSCO v. 4 (Simão *et al*. 2015; Waterhouse *et al*. 2018) with Viridiplantae and Eukaryote lineage dataset based on OrthoDB release 10. EnTAP (Hart *et al*. 2019) and Araport11 database (Cheng *et al*. 2016) was done for functional gene annotation.

### Genome annotation

Two hundred thirty-eight transcriptome libraries from Douglas-fir needles were used to improve genome annotation following the workflow (Supplementary Fig. 1e) available at Plant Genomics Lab's Gitlab. These were assembled into single-end SR (230 libraries, Cronn *et al*. 2017), paired-end SR (4 libraries, as described above), and Iso-Seq LR (4 libraries, as described above) *de novo* assembled transcriptomes and then clustered all together to reduce redundancy by identifying sequences that are at least 95% identical using VSEARCH v. 2.4.3 (Rognes *et al*. 2016).

The “transcriptome alignment” was generated by aligning the combined transcriptome to the Douglas-fir genome (Neale *et al*. 2017) using Gmap v. 2017-03-17 (Wu and Watanabe 2005) with the following parameters: -K 1000000, -L 10000000 –cross-species, –fulllength, –min-trimmed-coverage=.95, –min-identity=.95, and -n 1. Filtering followed using gFACs v 1.1.2 (Caballero and Wegrzyn 2019) with the parameters: –unique-genes-only, –min-CDS-size 300, –rem-genes-without-start-and-stop-codon, –allowed-inframe-stop-codons 0, –min-exon-size 9, and –min-intron-size 9.

HISAT2 v. 2.2.0 (Kim *et al*. 2019) was used to align all single-end SR (Cronn *et al*. 2017) and paired-end SR libraries (as described above) to Douglas-fir genome assembly (Neale *et al*. 2017). LR *de novo* transcriptome assembly was aligned to the genome using GTH v. 1.7.1 (Gremme *et al*. 2005). Both SR and LR alignments as well as protein alignments from NCBI RefSeq Plant Protein release version 87 and custom conifer geneset protein version 2 (available in Plant Genomics Lab's Gitlab) were provided as evidence to BRAKER2 (Hoff *et al*. 2016) to produce ab initio gene predictions, with the following parameters –prg = gth –gth2traingenes –softmasking 1 –gff3. Annotation v2 (prefilter) was generated after preliminary quality analysis of predicted genes using gFACs v. 1.1.2 with the following parameters: –min-CDS-size 300, –min-exon-size 9, –min-intron-size 9, –unique-genes-only, –rem-genes-without-start-and-stop-codon, and –rem-all-incompletes (Caballero and Wegrzyn 2019).

Gene models from Annotation v2 (pre-filter) were stringently filtered following the steps described below to produce a final high-quality annotation a.k.a. “Annotation v2.” Shorter BRAKER2 gene models were replaced with longer transcriptome alignment gene models using BEDtools v. 2.27.1 (Quinlan and Hall 2010). Gene models were further filtered based on repetitive content and annotated genes with sequences that were more than 80% softmasked in the genome were removed. Monoexonic genes and multiexonic genes were filtered based on functional annotation using EnTAP (Hart *et al*. 2019) similarity search with minimum target and query coverage set to 80% (also referred to as 80/80). The 50% coverage of the target and query sequence thresholds were also determined (also referred to as 50/50).

InterProScan v. 5.35-74.0 was run with the Pfam database (Hunter *et al*. 2009; Finn *et al*. 2016) to identify retrotransposons present in the putative gene model set. Genes labeled as “gag-polypeptide,” “retrotransposon,” “reverse transcriptase,” “retrovirus,” “copia,” or “gypsy” were removed. Monoexonic genes were further filtered ensuring they had valid Pfam domains based on the InterproScan output. The final gene set was functionally annotated using EnTAP (Hart *et al*. 2019), structurally assessed with gFACs (Caballero and Wegrzyn 2019), and evaluated for completeness with BUSCO (Simão *et al*. 2015).

### Genome annotation comparisons

We compared the quality of the Douglas-fir genome annotation models Annotation v2 (pre-filter), Annotation v2, published Douglas-fir genome annotation (a.k.a. “Annotation v1,” Cronn *et al*. 2017). Annotation v1 was executed with MAKER-P (v. 2.31.9) and derived from aligned transcripts published by Cronn *et al*. (2017) as well as protein evidence from publicly available gymnosperm transcriptomes (Neale *et al*. 2017). gFACs v. 1.1.2 (Caballero and Wegrzyn 2019) was used to gather preliminary statistics about each genome annotation. Completeness estimates for each model were produced by BUSCO v. 4.0.2 (Simão *et al*. 2015) based on single-copy orthologs in the lineage embryophyta_odb10. Completeness for combined SR and LR transcriptome and Annotation v2 were also determined using PLAZA coreGF v. 4.0 with green plant lineage as the primary reference (Van Bel *et al*. 2018). Each model was functionally annotated by running ENTAP v. 0.9.1 (Hart *et al*. 2019) on the proteins corresponding to the gene models (produced by gFACs) against NCBI's plant protein RefSeq database v. 87 (O’Leary *et al*. 2016) and a custom gymnosperm database composed of 186,061 sequences representing the proteomes of 7 species (*Picea abies, Picea sitchensis, Ginkgo biloba, Cycas micholitzii, Gnetum montanum, Taxus baccata*, and *Abies sachalinensis*). This was run twice for each annotation, with query and coverage set to 50/50 and 80/80, respectively.

## Results

### LR and SR transcriptome assembly

LR sequencing of 4 Douglas-fir RNA libraries representing control and stressed seedlings yielded a total of 1.75 million CCS (Table 1). After filtering, more than 700k reads with lengths greater than 50 bp were obtained from control and stressed samples. More than 90% of the filtered reads were FLNC reads. After clustering and polishing, 30k to 40k high-quality transcripts with lengths of 59 bp to 7.8 kbp were identified from each library. About 90% of the high-quality transcripts were identified as CDS with open-reading frames (ORF) and are likely protein-coding genes. Following these initial quality control steps, the number of unique transcripts obtained from stressed samples was similar to the number of unique transcripts obtained from control samples (Tables 1 and 2). The unique transcripts from the 4 libraries were combined to generate the *de novo* assembled LR transcriptome of Douglas-fir with 12,778 unique transcripts (NCBI TSA accession no. GISH00000000). The LR de novo assembled transcriptome was aligned to the Douglas-fir genome (Neale *et al*. 2017) and then collapsed to obtain unique transcripts only. This generated a reference genome-guided transcriptome assembly with only 9,611 unique transcripts (NCBI TSA accession no. GISF00000000). A total of 7,761 unique transcripts were common to both assemblies.

**Table 1.** Summary statistics for Iso-Seq LR sequencing libraries generated from needles of 4 Douglas-fir plants. Libraries were obtained from 2 control and 2 stressed plants. Number of CCS reads, filtered reads with length greater than 50 bp, FLNC reads, and high-quality transcripts and unique transcripts are shown.

				High-quality transcripts				Unique transcripts			
Treatment	CCS No.	Filtered reads No.	FLNC No.	No.	Min. (bp)	Max. (bp)	Ave. (bp)	No.	Min. (bp)	Max. (bp)	Ave. (bp)
Control	439,695	378,572	365,857	36,833	69	7,843	1,869	10,852	270	6,855	1,316
Control	412,286	345,219	315,096	31,426	65	6,549	1,608	9,036	273	5,112	1,189
Stressed	472,728	406,259	394,753	40,418	59	7,647	1,898	10,012	297	5,232	1,306
Stressed	426,646	376,543	372,177	40,536	64	6,711	1,959	9,374	270	5,001	1,353

The same RNA extractions that were used for the LR sequencing described above were also used in a parallel SR sequencing approach to create an SR *de novo* transcriptome assembly. We obtained a highly variable number of raw reads from each of the 4 libraries ranging from 90 to 206 million (Table 2). Clustering of all 4 SR libraries generated an SR *de novo* transcriptome assembly with 142,381 unique transcripts and 37,011 unique transcripts with ORFs. As expected, the number of transcripts assembled from SR sequencing was greater than the number of transcripts obtained for the LR transcriptome. Interestingly, the longest transcript was assembled from SR data and not LR.

**Table 2.** Summary statistics for NovaSeq SR RNA sequencing libraries generated from needles of 4 Douglas-fir plants. A total of four libraries were obtained from 2 control and 2 stressed plants. Number of raw reads, trimmed reads, assembled reads, and unique transcripts are shown.

			Assembled reads				Unique transcripts			
Treatment	Raw reads No.	Trimmed reads No.	No.	Min (bp)	Max (bp)	Ave (bp)	No.	Min (bp)	Max (bp)	Ave (bp)
Control	103,224,378	101,124,225	107,557	351	14,605	1,524	33,902	258	13,227	987
Control	206,055,811	201,383,972	129,103	351	14,246	1,395	36,304	255	13,524	931
Stressed	174,962,423	170,250,648	119,558	351	18,854	1,577	36,302	255	16,926	977
Stressed	91,600,485	89,524,883	95,477	351	20,394	1,559	30,918	261	12,441	996

The total number of LR-generated unique transcripts in control and stressed samples was 10,046 and 10,734, respectively (Tables 1 and 2). These values were 2.8× less than the unique transcripts generated using de novo SR assembly. About 80% of unique transcripts in the control treatment had identical sequences to stressed samples.

### Assembly and comparison of LR *de novo* and reference genome-guided transcriptome assembly

**Fig. 1.**
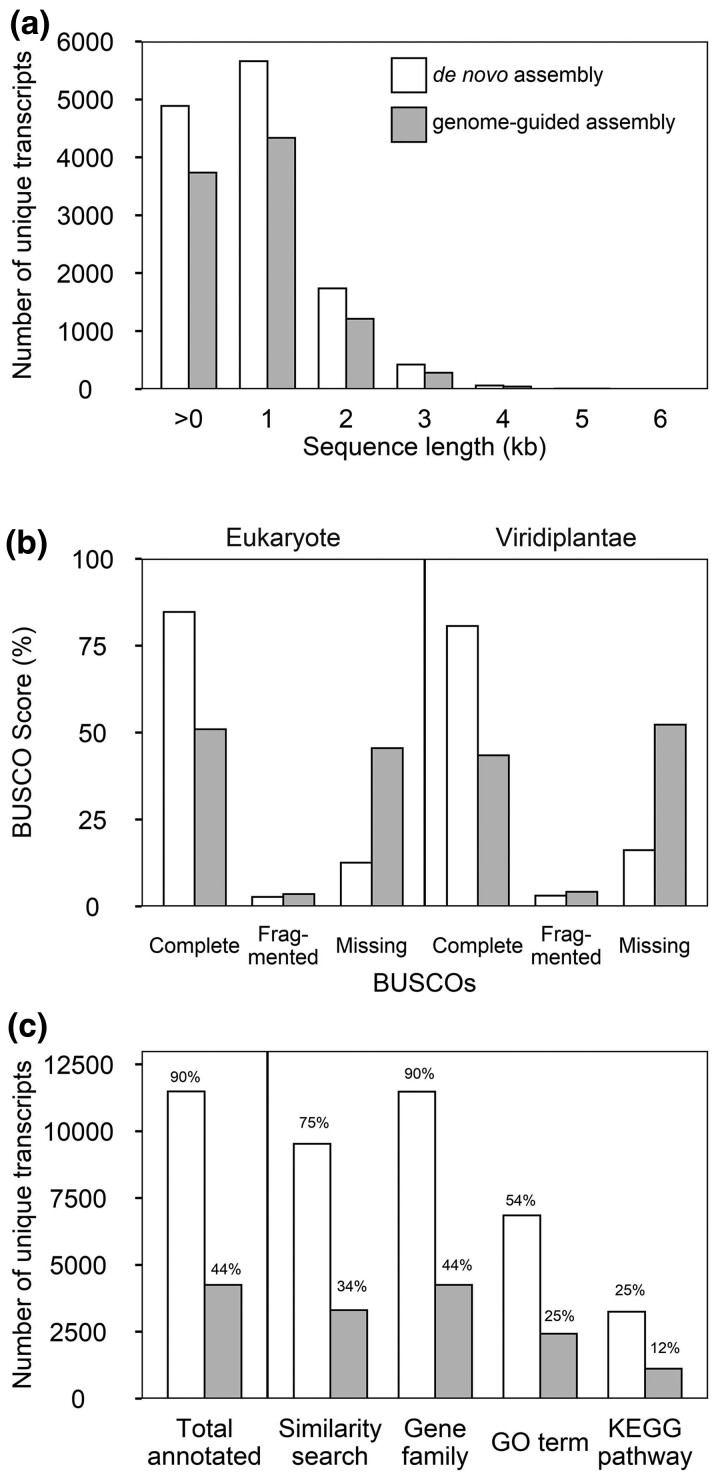
Comparison of quality of *de novo* and reference genome-guided assembly of Douglas-fir LR-generated transcriptome. a) Length versus number of unique transcripts and b) transcriptome completeness score. c) Number and percentage of unique transcripts with functional annotation.

**Fig. 2.**
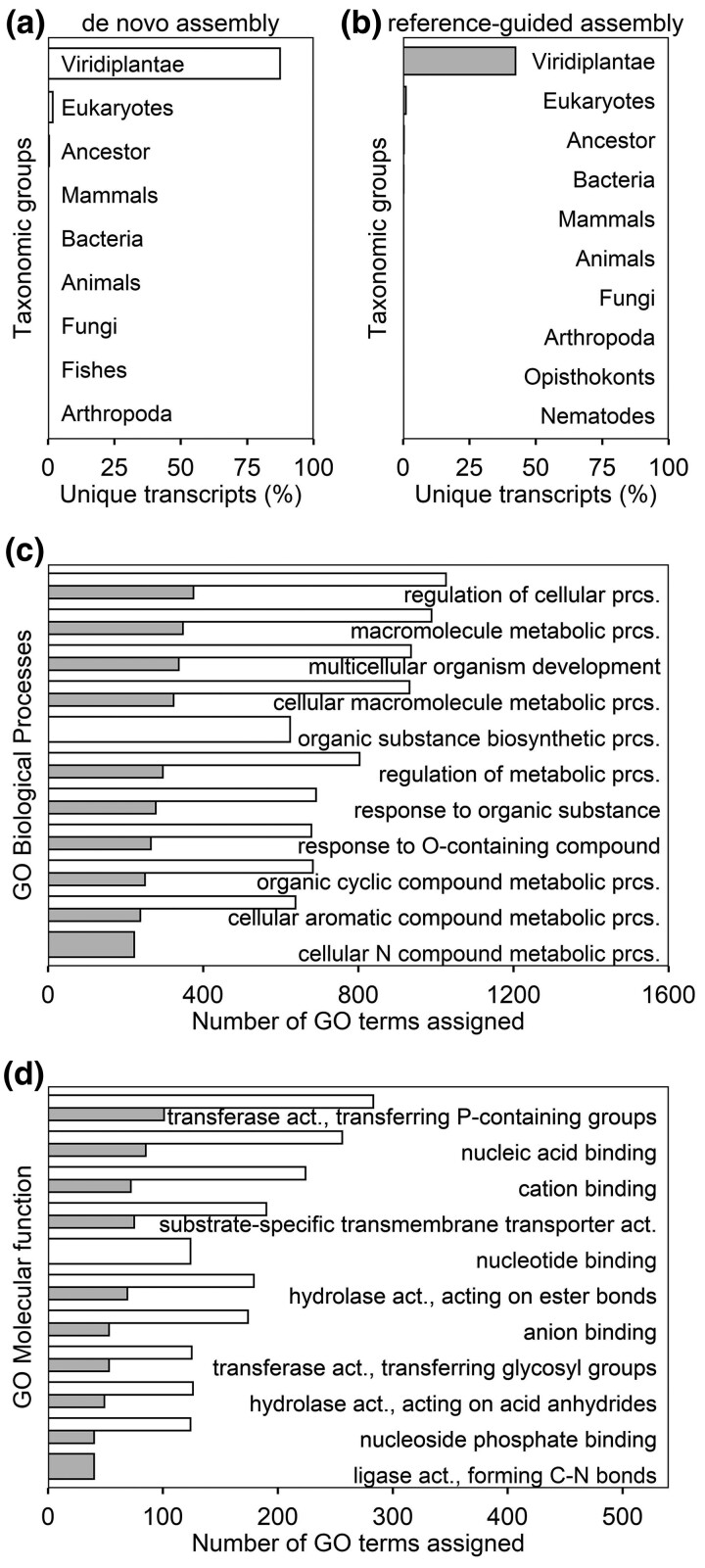
Summary of gene family and gene ontology assignments in de novo assembled Douglas-fir LR transcriptome. Top ten a,b) taxonomic groups versus unique transcripts, c) GO biological processes, and d) GO molecular function terms versus number of GO terms assigned. White and gray bars represent data from de novo and genome-guided transcriptome assembly, respectively.

The quality of *de novo* and reference genome-guided LR assemblies was assessed by quantifying the length of transcripts, completeness, and a number of unique transcripts with functional annotation (Fig. 1) including taxonomic group and GO terms assignments (Fig. 2, Supplementary File 1, 2).

The *de novo* assembled LR transcriptome had more and longer transcripts compared to the genome-guided LR assembly (Fig. 1a). The N50 value for *de novo* LR assembly was 3,150 bp which is 420% greater than the N50 value for the genome-guided LR assembly.

The transcriptome completeness of our assemblies was quantified with BUSCO (Simão *et al*. 2015; Waterhouse *et al*. 2018; Fig. 1b). We searched a total of 425 BUSCO groups in Viridiplantae dataset and 255 BUSCO groups in Eukaryote dataset. The *de novo* LR assembly of Douglas-fir transcriptome has a complete BUSCO score of 80.7% and 84.7% using Viridiplantae and Eukaryote lineages, respectively. The BUSCO scores obtained for the *de novo* transcriptome assembly using the LRs demonstrated that the LR *de novo* assembled transcriptome contains almost twice as many complete single-copy orthologs compared to the genome-guided LR assembly which only has 185 (43.5%) of BUSCO genes present from the Viridiplantae lineage database. Both *de novo* and genome-guided LR transcriptome had very few fragmented BUSCOs (13 and 18, respectively) with values below 2% of total BUSCO. However, the number of missing orthologs and highly incomplete/fragmented transcripts was much higher as captured by the number of missing BUSCO groups. There are 222 missing BUSCOs in genome-guided and only 69 in *de novo* LR transcriptome.

The sequence similarity and gene family assignment of our LR assemblies were assessed using the *Arabidopsis* dataset (Fig. 1c). This generated functional annotations for 11,490 out of 12,778 (90%) unique transcripts in the LR *de novo* assembly. About 54% and 25% of the unique transcripts in the *de novo* assembly have GO term and KEGG pathway assignments, respectively. The equivalent values for the LR reference-guided assembly were less than half of the annotation statistics for the *de novo* approach.

In both the *de novo* and genome-guided LR transcriptomes, most of the unique transcripts with gene family assignment belonged to Viridiplantae taxa, and a small fraction was assigned broadly to Eukaryotes (Fig. 2, a and b). That is, 87% or 11,168 and 42% or 4,072 unique transcripts in *de novo* and genome-guided LR transcriptomes, respectively, were assigned to Viridiplantae taxa. Exactly 6,853 unique transcripts were assigned to at least one GO term with a total of 261,256 GO terms in the *de novo* LR assembly. A total of 4,322 unique biological GO terms were assigned of which regulation of cellular process (GO:0050794), several metabolic processes (GO:0043170, GO:0044260, GO:0019222, GO:1901360, GO:0006725), multicellular organism development (GO:0007275), response to O-containing compound (GO:1901700), and organic substance biosynthetic process (GO:1901576) had the most unique transcripts assigned (Fig. 2c). Only 1,770 unique molecular function terms were assigned including those pertaining to transferase (GO:0016772 and GO:0016757) and hydrolase activity (GO:0016788 and GO:0016817; Fig. 2d). On the other hand, the LR transcriptome assembly conducted with the reference genome generated 2,245 unique transcripts with at least one GO term and a total of 92,372 GO terms assigned. Despite fewer GO annotations, the top 10 GO biological process terms (Fig. 2c) and molecular function terms (Fig. 2d) observed for genome-guided LR transcriptome assembly were similar to the LR *de novo* transcriptome assembly.

We also queried the quality of the SR *de novo* transcriptome assembly. The SR transcriptome has an almost perfect complete BUSCO with 421 complete BUSCOs out of 425 (99.1%) total BUSCOs on Viridiplantae lineage despite a shorter N50 value of 1,878 bp. We also found a high percentage of functional annotation with 63% or 23,334 out of 37,011 unique transcripts in SR *de novo* transcriptome. However, only 11,932 transcripts were annotated with at least one GO term, and only 5,108 has KEGG annotation. The proportion of SR *de novo* transcripts annotated with GO (32%) or KEGG (13%) terms is similar to genome-guided transcriptome assembly and much lower than LR *de novo* assembly (Fig. 1c).

### Prediction of TF and TF domain catalog

We identified 2,016 putative TFs in our *de novo* LR assembly (Table 3, Supplementary Table 1). The putative TFs were classified under superclass basic domain (269 unique transcripts), zinc-coordinating DNA-binding domain or zinc finger (518 unique transcripts), helix-turn-helix (722 unique transcripts), beta-scaffold factors (374 unique transcripts), and other (133 unique transcripts). Only 613 of the putative TFs had a known DNA-binding motif.

**Table 3.** Number of putative TF in LR de novo transcriptome assembly and classification.

TransFac TF classification		Unique transcripts
Code	Classification	No.
0.0.0.0.0	Other	104
0.5.1.0.1	Other, AP2-related factors	29
1.0.0.0.0	Basic domain	239
1.1.0.0.0	Basic domain, Leucine zipper factors (bZIP)	30
2.0.0.0.0	Zinc finger	518
3.0.0.0.0	Helix-turn-helix	689
3.1.0.0.0	Helix-turn-helix, homeo domain	33
4.0.0.0.0	Beta scaffold	373
4.10.0.0.0	Beta scaffold, cold shock domain factors	1

We cross-referenced the putative TFs against the functional annotation performed earlier (Supplementary File 2). We identified 1,466 out of 2,016 putative TFs. Only 402 out of 613 putative TF with known DNA-binding motif had orthologous genes in *Arabidopsis*. We observed that 13% of putative TFs belong to tetratricopeptide repeat (TPR)-like, pentatricopeptide repeat, NAD(P)-binding Rossmann-fold, ARM repeat, and MYB superfamily proteins. We also compared the protein sequence of putative TFs to the Plant Transcription Factor Database v5.0 (PTFDB) for Douglas-fir which contains 1,915 TFs (Jin *et al*. 2017). We identified 1,536 TFs in Douglas-fir PTFDB that were similar to 411 of the putative TFs predicted by TFPredict (Supplementary Table 2).

### Identification of lncRNA

**Fig. 3.**
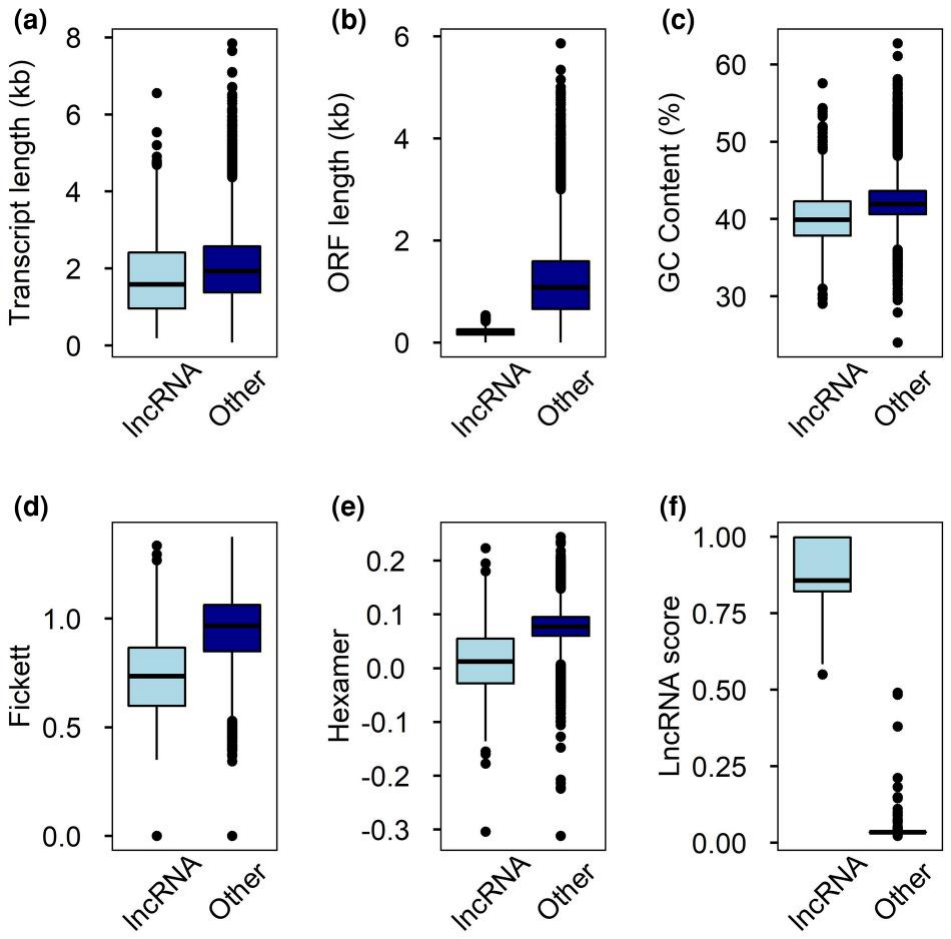
Trait values of transcripts predicted as lncRNAs in Douglas-fir. a) Mean transcript length, b) ORF length, c) GC content, d) Fickett testscore, e) Hexamer score, and f) lncRNA score of lncRNAs and all other assembled transcripts including protein-coding transcripts.

We identified a total of 666 putative lncRNAs from the 14,783 unique polished transcripts derived from our LR sequencing data (Fig. 3, Supplementary File 3). The lncRNA transcript lengths ranged between 184 bp and 6,549 bp (Fig. 3a). We found 73% of the Douglas-fir lncRNAs were larger than 1 kb and all had ORF length lower than 1 kb (Fig. 3b). Lower Fickett testscore and hexamer score indicate lower coding potential for predicted lncRNAs (Fig. 3, d and e). Using BLASTN and the RNACentral (The RNAcentral; Consortium *et al*. 2019) *Arabidopsis* lncRNA database, we identified orthologs for 6 putative lncRNA in Douglas-fir (Supplementary Table 3). A comparison with the complete noncoding RNA (ncRNA) database from RNAcentral Release 14 provided a total of 62 significant alignments (Supplementary Table 4). From the predicted Douglas-fir lncRNAs, 14 were known Douglas-fir ncRNA, and several were orthologous to ncRNA described in conifers including spruce (*Picea* spp., 31), pine (*Pinus* spp., 3), fir (*Abies* spp., 2) and 2 conifer species native to Asia (*Dacrycarpus imbricatus* and *Cathaya argyrophyll*a). Nine putative lncRNAs from Douglas-fir have orthologs in other plants including *Arabidopsis*, barrel clover (*Medicago tranculata*), and rubber tree (*Hevea brasiliensis*). Only 21 of the predicted Douglas-fir lncRNAs were assigned to a GO term. Seven transcripts were assigned to at least one GO biological process term, e.g. intron splicing (GO:0000372 and GO:0000373), gene silencing (GO:0035195), and RNA catabolic process (GO:0006401). Fourteen lncRNAs were assigned to GO molecular function terms triplet codon-amino acid adaptor activity (GO:0030533) and GO cellular component term ribosome (GO:0005840). We also found 4 Douglas-fir lncRNAs assigned to a structural constituent of ribosome (GO:0003735).

### Improved genome annotation

**Fig. 4.**
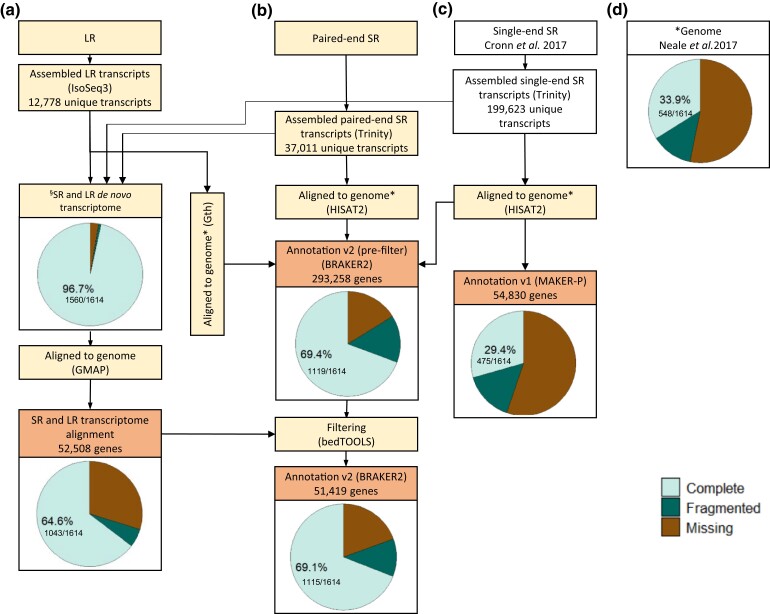
Genome annotation processes and BUSCO completeness. (a–c) Flow chart summarizes the steps taken to obtain genome annotations (b) Annotation v2 (pre-filter) and Annotation v2 and (c) Annotation v1. (d) BUSCO scores for genome assembly* used in genome annotation steps and *de novo* assembled transcriptome^§^ are also shown.

The LR and paired SR *de novo* transcriptomes assembled here were combined with Cronn *et al*.'s (2017) unpaired SR transcriptome assembly to assess the potential for improved annotation of the Douglas-fir genome. Statistics describing the assembled SR and LR *de novo* transcriptome aligned to genome (SR and LR transcriptome alignment), published genome annotation (Annotation v1; Cronn *et al*. 2017; Neale *et al*. 2017), genome annotation generated using BRAKER2 before refinement [Annotation v2 (pre-filter)] and after refinement by re-integrating transcriptome data (Annotation v2) are provided in Table 4 and Supplementary Table 5. A summary of the steps taken to generate an improved genome annotation with BUSCO scores is shown in Fig. 4.

**Table 4.** Genome annotation summary statistics.

	a) SR and LR transcriptome alignment	b) Annotation v1	c) Annotation v2 (pre-filter)	d) Annotation v2
Total genes	52,508	54,830	293,458	51,419
Average gene length (bp)	27,489.02	9,011.77	4,933.09	17,967.11
Median gene length (bp)	2,019	2,571	633	1,962
Multiexonics	38,430	47,874	111,981	41,595
Monoexonics	14,078	6,956	181,477	9,824
Longest intron (kb)	778,429	269,672	256,822	778,429
Average number of exons per multiexonic gene	5.38	4.86	3.55	4.73
Functional annotation (50/50) %	72.50%	81.87%	33.80%	100%

The SR and LR transcriptome alignment accounted for a total of 52,508 genes with 14,078 aligning as monoexonic and 38,430 as multiexonic (Table 4a, Fig. 4a). None of the aligned genes from the assembled LR transcripts were missing in the SR transcriptome. About 80.9% of the genes were from the assembled paired-end SR transcripts and 19.1% were from the assembled single-end SR transcripts.

The resulting annotation generated by BRAKER2 and subsequent transcriptome alignments was labeled Annotation v2 (pre-filter), and resulted in 293,458 genes with 181,477 monoexonic and 111,981 multiexonic genes (Table 4c, Supplementary Table 5, Fig. 4b). The annotation reflects *ab initio* predictions from the full set of RNA SR alignments and protein alignments of the translated LR transcripts. The *ab initio* predictions were supplemented with transcriptome alignments that represented all *de novo* assembled inputs. Further refinement via filtering based on functional annotation, repetitive content, and the transcriptome alignment resulted in Annotation v2's final gene count of 51,419 genes with 9,824 monoexonic and 41,595 multiexonic genes (Table 4d, Supplementary Table 5, Fig. 4b). About 94.7% of the genes in Annotation v2 were also found in the prefiltered set, and rest were derived exclusively from the transcriptome alignment (none from LR, 2,053 paired-end SR, and 629 from single-end SR transcriptome). The total gene count in Annotation v2 was only 6% less than in Annotation v1 which has 54,830 genes (6,956 monoexonic and 47,874 multiexonic; Table 4b, Supplementary Table 5, Fig. 4c). Filtering efforts were focused on the reduction of a large number of false positives predicted as monoexonic genes. The most significant decrease for Annotation v2 was seen in filtering based on the presence of a functional annotation at the 50/50 query coverage level which removed over 148,000 genes.

BUSCO scores were also variable in the transcriptomes and genome annotations (Fig. 4). The SR and LR transcriptome alignment was 64.6% complete in comparison to the full set SR and LR *de novo* transcriptome which was 96.7% complete (Fig. 4a). The lower completeness score in SR and LR transcriptome alignment relative to SR and LR *de novo* transcriptome was coupled with more than 7.5X and 11.4X increase in fragmented and missing BUSCOs, respectively. Annotation v2 was 69.1% (Fig. 4b) complete, a significant improvement from the published set of models (Cronn *et al*. 2017; Neale *et al*. 2017) in Annotation v1 which was 29.4% complete as assessed by BUSCO using 1614 total BUSCO groups (Fig. 4c). There was a slight decrease in completeness through the filtering process from 69.4% to 69.1%; however, this was paired with the removal of over 200,000 unlikely models.

**Fig. 5.**
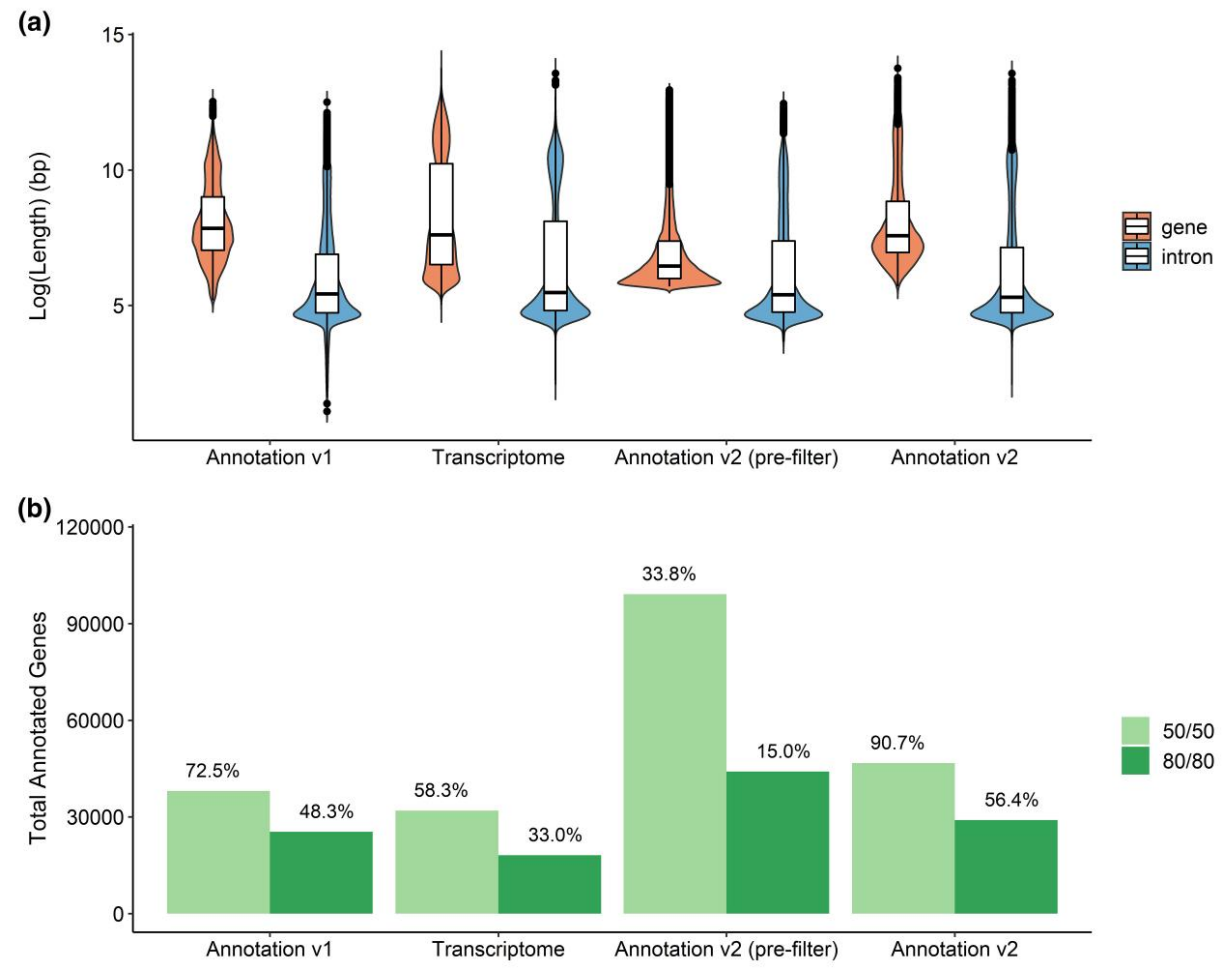
Genome annotation evaluation. a) Gene and intron length distribution across genome annotation approaches, and the transcriptome alignment (*de novo* assembled prior to alignment). The log-scaled values for gene length and intron length reflect improvements in contiguity with the addition of Iso-Seq data. b) Reciprocal BLAST-style analysis was conducted at 2 coverage values for the total set of genes produced from each method. The 50% coverage of the target/query and 80% coverage of target and query are shown. The numerical value at the end of each bar represents the percentage of total sequences that were functionally annotated at that coverage value.

Both intron and gene lengths improved significantly in Annotation v2 when compared to the published models (Fig. 5a). Annotation v2 had a maximum intron length of 778 kbp, which was significantly longer than the longest intron in Annotation v1 at 269 kbp. The detection of massive introns indicates an improvement in the annotation quality, as long introns are characteristic of conifer species (Nystedt *et al*. 2013). Additionally, Annotation v2 had a longer average gene length at 17.97 kbp compared to 9.01 kbp in Annotation v1 (Fig. 5, Table 4, Supplementary Table 5). This is comparable to transcriptome alignment which has an average gene length of 27 kbp. The additional step of re-integrating the SR and LR transcriptome alignments was responsible for extending a total of 2,061 BRAKER2 gene models with as many as 20 genes spanned by a single transcript alignment. By identifying overlapping regions between the annotation and the transcriptome alignment, putative gene models that were completely nested within high-quality transcriptome alignments were removed. Partially overlapping gene models from the final set were resolved by selecting the longer and more complete gene model.

The number of functionally annotated genes also increased in Annotation v2 to 100% with 50/50 query/target threshold levels (Fig. 5b). All 51,419 genes in Annotation v2 had functional annotations. Of these, 29,001 (56.4%) were annotated at the more stringent 80/80 query/target threshold against the same databases (Fig. 5b). This was an improvement from Annotation v1 which reported only 47.3% functionally annotated genes at the 80/80 coverage threshold.

## Discussion

### Comparison of Douglas-fir de novo and genome-guided transcriptome assembly

This study examined the transcriptome assemblies (genome-guided and *de novo* constructed) across a plethora of quantitative metrics to determine that the *de novo* assembly was a far more accurate approach (Fig. 1). Despite the moderately high contiguity and estimated completeness in terms of genome size of the Douglas-fir genome reference, the fragmentation clearly remains problematic in genic regions. The resulting *de novo* LR transcriptome was nearly twice as complete (BUSCO) and of significantly higher quality in terms of overall length and resolution of FL ORFs (Fig. 1b). The genome was unable to provide a benefit beyond what was achieved through the resolution of the LR independently.

Moreover, 75% of the unique high-quality Iso-Seq transcripts that aligned to the Douglas-fir reference genome (Fig. 1a) is consistent, in terms of percentage, with other more fragmented conifer genome assemblies, such as sugar pine (60%; Gonzalez-Ibeas *et al*. 2016). This is not the case for more complete conifer genomes, such as the recently assembled, chromosome-scale giant sequoia genome, that reported alignment rates over 80%.

### Transcriptome atlas of Douglas-fir using de novo assembly approach

We present an LR *de novo* transcriptome assembly of Douglas-fir, a comprehensive and high-confidence set of transcripts for Douglas-fir with an 80.7% complete BUSCO score and 90% functionally annotated transcripts (Fig. 1). This is comparable to the published sugar pine Iso-Seq derived transcriptome assembly (78% complete and 93% annotated; Gonzalez-Ibeas *et al*. 2016). This is a huge leap forward when compared to 19% (Hess *et al*. 2016) or 27% of identified unique transcripts from early Douglas-fir needle transcriptomes with functional annotation based on *Arabidopsis thaliana* database (Müller *et al*. 2012). This can also be seen as an improvement over the completeness of the primary transcriptome resource for the first version of the Douglas-fir genome annotation (Cronn *et al*. 2017) at 85.6% complete and 12% with functionally annotated. The first metric demonstrates the benefit of very deep sequencing since Cronn et al.’s (2017) study included 179 needle libraries across 24 timepoints to examine diurnal and circannual gene expression variation in Douglas-fir. The latter metric reflects the fragmentation of the transcriptome assembly that results from assembling single-end SRs.

When combined with the SR resources, the Douglas-fir transcriptome (i.e. SR and LR transcriptome) is nearly fully complete with 97% complete BUSCOs (Fig. 4) and 92% complete based on PLAZA coreGF. The published LR/SR transcriptome study in sugar pine, and the data here from Douglas-fir, strongly support the value of combining both LR and SR data sets to achieve a more comprehensive view of the gene space. While SR assembly is challenged by the nature of the SRs, deep sequencing can resolve more of the expressed rare isoforms (Gonzalez-Ibeas *et al*. 2016). This is further supported by the giant sequoia transcriptome which achieved 20% BUSCO completeness from the Iso-Seq transcripts alone (through moderate depth sequencing) compared with 81% when combined with the SR-derived transcriptomes (Scott *et al*. 2020). Here, as in most cases, the LRs provide specific value in validating monoexonics as well as resolving splice variants. While several challenges still exist in resolving consistent gene annotations directly from LR data, their role is clearly supported for high-quality transcriptome catalogs (Chow *et al*. 2019; Feng *et al*. 2019; Kuo *et al*. 2020). In the LR *de novo* transcriptome, we identified more than 2,000 putative TFs in Douglas-fir of which only 30% have a known DNA-binding domain (Table 3). The number of putative TFs that we identified is lower by 682 when compared to the annotated TFs in the Douglas-fir genome (Neale *et al*. 2017). PTFDB's TF list is only 5% shorter than our list, however, BLASTn identified 80% of PTFDB's TFs correspond to 20% of the TFs we predicted suggesting that PTFDB's lists unique TFs more than once. This is not surprising given the higher total unique transcripts identified for Douglas-fir in published assemblies and may be a result of annotation errors. Among Douglas-fir TFs were orthologs of well-studied TFs in *Arabidopsis*. Examples are *Arabidopsis* orthologs for *TPR*, which codes for a group of proteins known to be involved in cellular functions and which are essential in responses to hormones such as ethylene, cytokinin, gibberellin, and auxin (Schapire *et al*. 2006). Some like *Arabidopsis TOC64* and spinach *TCP34* are suggested to be involved in biogenesis of photosynthetic apparatus (Bohne *et al*. 2016). Interestingly, we found 2 copies of *TOC64* in Douglas-fir (Locus IDs transcript9626.p1_1, transcript10803.p1_1) both with about 89% coverage and greater than 50% identical to *Arabidopsis* TOC64 (Locus ID *AT3G17970.1*). We found many other Douglas-fir orthologs to *Arabidopsis* genes important in photosynthesis including *Arabidopsis High Chlorophyll Fluorescent 107*, *TCP34*, *Pyg7, LPA1, MET1,* and *FLU*. We also identified *Arabidopsis* orthologs encoding for enzymes such as hydrolases, epimerases, kinases, and phosphatases. Since Douglas-fir TF repertoire is mostly unexplored experimentally, we reckon that DNA-binding motif validation through chromatin immunoprecipitation (ChIP)-PCR should be done prior to exclusion of any suspicious putative TFs.

### LncRNA catalog

This is the first study on lncRNA in Douglas-fir and one of few in conifers (Nystedt *et al*. 2013; Liu and El-Kassaby 2019). The number of lncRNA predicted in our study for Douglas-fir is 666. This is low compared to 3,887 predicted lncRNAs in *Arabidopsis* (The RNAcentral Consortium *et al*. 2019), 1,187 lncRNAs in poplar (Chao *et al*. 2019), 2,044 lncRNAs in gingko (Wu *et al*. 2019), or 9,686 lncRNAs in spruce (Nystedt *et al*. 2013). The low number of putative lncRNAs identified in Douglas-fir is likely the consequence of the combined use of the conservative CREMA lncRNA prediction tool with the ensemble model trained on experimentally validated lncRNAs only (Simopoulos *et al*. 2018). As expected, we identified only a handful Douglas-fir lncRNAs which were homologous to other plant species (Supplementary Tables 3 and 4) due to the inherent poor sequence conservation of this RNA class across species (Ponjavic *et al.* 2007; Johnsson *et al*. 2014).

### Improved genome annotation

Significant improvements in completeness, functional annotation, and fragmentation were observed in the updated annotation, presented here as Annotation v2. The published genome annotation (Annotation v1) was produced using MAKER-P which incorporated evidence from assembled unpaired SR transcripts (Cronn *et al*. 2017; Neale *et al*., 2017). We compared Annotation v1 to Annotation v2 which was composed of BRAKER2 ab initio gene predictions trained by paired-end and single-end SR data, translated LR transcripts provided as protein alignments, and gene models derived from the aligned transcriptome (also referred to as “SR and LR transcriptome alignment”).

While the base genome estimate of completeness is shockingly low at 33.9%, this can be attributed to the poor performance of benchmarking tools like BUSCO when spanning large regions of intronic space (Fig. 4). The most comparable statistics are between the aligned transcripts (assessed as proteins) and the final improved gene models. Here, we note that the transcriptome, composed of both SR and LR-derived assemblies, is nearly complete on its own with a 96.7% complete BUSCO score (and 92% complete based on PLAZA coreGF). When aligned to the genome, we recover 64.6% of single-copy orthologs. This discrepancy is likely the result of fragmentation that remains in the source reference assembly.

Annotation v2 which is just under 70% complete is the best genome annotation presented here. This improvement reflects on the implementation of a new informatics pipeline that extends on BRAKER2, integration of LR transcripts within ab initio prediction, and extensive downstream filtering to contend with the large quantity of false-positive identifications.

The high heterozygosity, ploidy and copy repeats, and prevalent pseudogenes and transposable elements in a plant mega-genome complicate assembly and often result in high fragmentation (Schatz *et al*. 2012). As such, a combination of multiple approaches to filter out false gene models was required to improve this annotation. Mono/multiexonic ratios, transposable elements, and pseudogenes dominated the over 290k genes initially generated from ab initio prediction. The ab initio component allowed for the identification of genes not represented in the aligned transcriptome and aligned transcripts improved the quality of initial BRAKER2 models when used again following the first round of prediction. It should be noted that the aligned LR transcripts did not extend the predicted models from BRAKER and any extensions were the result of the new SR transcripts. This may be a factor of slightly inflated error rates in the final transcripts not reflected in the Illumina SR transcripts since very stringent filters are used to accept aligned transcripts as true gene models. The aligned LR transcripts did, however, contribute as aligned protein models to train and improve the ab initio approach. In this sense, the protein evidence can correct or resolve intron/exon boundaries and correct initial predictions from SR data alone. It should be noted that this approach does not consider more complex models that can weigh evidence across prealigned and predicted models (http://eugenes.org/EvidentialGene/). Overall, the BUSCO benchmark score reports the final annotation as nearly 70% complete which is far favorable to the 29% seen in Annotation v1. Annotation v2's complete BUSCO score is similar with PLAZA coreGF's completeness estimation for this annotation at 71.5%. This high completeness for Annotation v2 is complemented by the results of the in-depth functional characterization performed on the final models. The reciprocal BLAST-style analysis noted that all final models aligned over at least 50% of their length and the corresponding target sequence (Fig. 5b).

The power of FL, high-quality transcripts can also be seen in the structural characteristics of the final models. This includes gene length, CDS length, splice sites, and identification of both start and stop codons in the final models. The average length of the final genes increased by more than 2-fold (Fig. 5a). All accepted genes were completed with start and stop positions. Long introns characteristic of conifer genomes were maintained. The longest intron, at 778 kbp, was supported by a transcriptome alignment that would otherwise have gone undetected from the BRAKER2 process alone. Improvement of gene length was also reflected in the monoexonic/multiexonic ratio which is a tremendous challenge in genomes that are as repetitive as conifers (>80%; Kovach *et al*. 2010; Mosca *et al*. 2019). The ability to distinguish pseudogenes remains challenging and is the source of the high number of false positives produced by nearly all gene annotation approaches (Nystedt *et al*. 2013). Standard informatic filters inadvertently remove true genes or leave in too many pseudogenes. A complete and high-quality transcriptome was crucial to resolve this in Douglas-fir.

Relative to published conifer genome annotations, this genome annotation is comparable to the best conifer genome available today, both in terms of statistics of lengths and completeness. The chromosome-scale giant sequoia genome provides a completeness estimate of 68.96% for its gene space (Scott *et al*. 2020). The new Douglas-fir genome annotation exceeds gene space estimates for all other public annotations including *Pinus lambertiana* (40.5%), *Pinus taeda* (37.8%), *Abies alba* (15.8%), and *Picea abies* (28.1%; Nystedt *et al*. 2013; Stevens *et al*. 2016; Zimin *et al*. 2017; Mosca *et al*. 2019).

## Supplementary Material

jkac304_Supplementary_Data

## Data Availability

LR raw reads (accession nos. SRR12208323 to SRR12208326), SR raw reads (accession nos. SRR12208319 to SRR12208322), and assembled sequences (GISH00000000 and GISF00000000) generated from Douglas-fir BioSamples (accession nos. SAMN15501818 to SAMN15501821) are available under NCBI BioProject ID PRJNA614528. BioSamples are from a total of 4 individual Douglas-fir plants exposed to control and stress treatments as described under *Plant Material*. Douglas-fir genome annotation is available at TreeGenes (treegenesdb.org/FTP/Genomes/Psme/v1.0/). Custom conifer geneset used for genome annotation is available in Plant Genomics Lab's Gitlab (https://gitlab.com/PlantGenomicsLab/HQ_Douglas-fir_transcriptome_genome_annotation). Supplemental material available at G3 online.

## Notes

*Communicating editor: J. Holland*
