# Supplementary material for: A long-read and short-read transcriptomics approach provides the first high-quality reference transcriptome and genome annotation for Pseudotsuga menziesii (Douglas-fir)
Source: G3 (Bethesda). 2022 Dec 1;13(2):jkac304. doi: 10.1093/g3journal/jkac304 (PMC10468028; doi:10.1093/g3journal/jkac304)
Supplement: jkac304_Supplementary_Data [file jkac304_supplementary_data.zip › Supplemental_Material_Legends_G3-2022-403847.docx]

## Supporting materials

**Table S1. Putative transcription factors were identified from Douglas-fir *de novo* transcriptome assembly.** The table shows the Locus ID of unique transcripts identified as putative transcription factors as well as their TransFac classification and InterPro DNA motif accessions, types,and names.

**Table S2. Douglas-fir putative transcription factors present in our LR *de novo* assembly and PTFDB.** BLASTp results with PTFDB as query sequence and LR *de novo* assembly as subject sequence. The *E*-value cutoff was set to 1E-5.

**Table S3. Putative lncRNA in Douglas-fir with *Arabidopsis* orthologs.** RNACentral lncRNA database was searched for *Arabidopsis* orthologous lncRNA to Douglas-fir putative lncRNAs. BLASTN was used at an *E*-value cutoff of 1E-5.

**Table S4. Putative lncRNA in Douglas-fir with high similarity to known non-coding RNAs.** Sequences of Douglas-fir putative lncRNAs were compared to sequences of known non-coding in RNACentral release 14. BLASTN was used at an *E*-value cutoff of 1E-5.

**Table S5. Full gFACs Statistics for Each Annotation Model.** Full summary statistics produced by gFACs for the finalized version of each model: Annotation v1, Transcriptome alignment, Annotation v2 (pre-filtering), and Annotation v2.

**Figure S1. High-quality transcriptome and genome annotation workflow.** (A) Iso-Seq LR data quality control and transcriptome assembly. (B). Identification of Transcription Factors from Iso-Seq LR data. (C) Identification of long non-coding RNA from Iso-Seq LR data. (D) Illumina SR quality control and transcriptome assembly. (E) Genome annotation. (F) Work performed by Cronn et al. 2017 (black text on white background). Light gray background is used to distinguish each section of the workflow. Genome annotation models were labeled with black text on orange background.

**File S1. Functional annotation of Douglas-fir transcripts from reference-guided transcriptome assembly.**

**File S2. Functional annotation of Douglas-fir transcripts from *de novo* transcriptome assembly.**

**File S3. Sequences of putative long non-coding RNA identified from Iso-Seq unique transcripts.**
