# Supplementary figures and images for: A long-read and short-read transcriptomics approach provides the first high-quality reference transcriptome and genome annotation for Pseudotsuga menziesii (Douglas-fir)
Source: G3 (Bethesda). 2022 Dec 1;13(2):jkac304. doi: 10.1093/g3journal/jkac304 (PMC10468028; doi:10.1093/g3journal/jkac304)

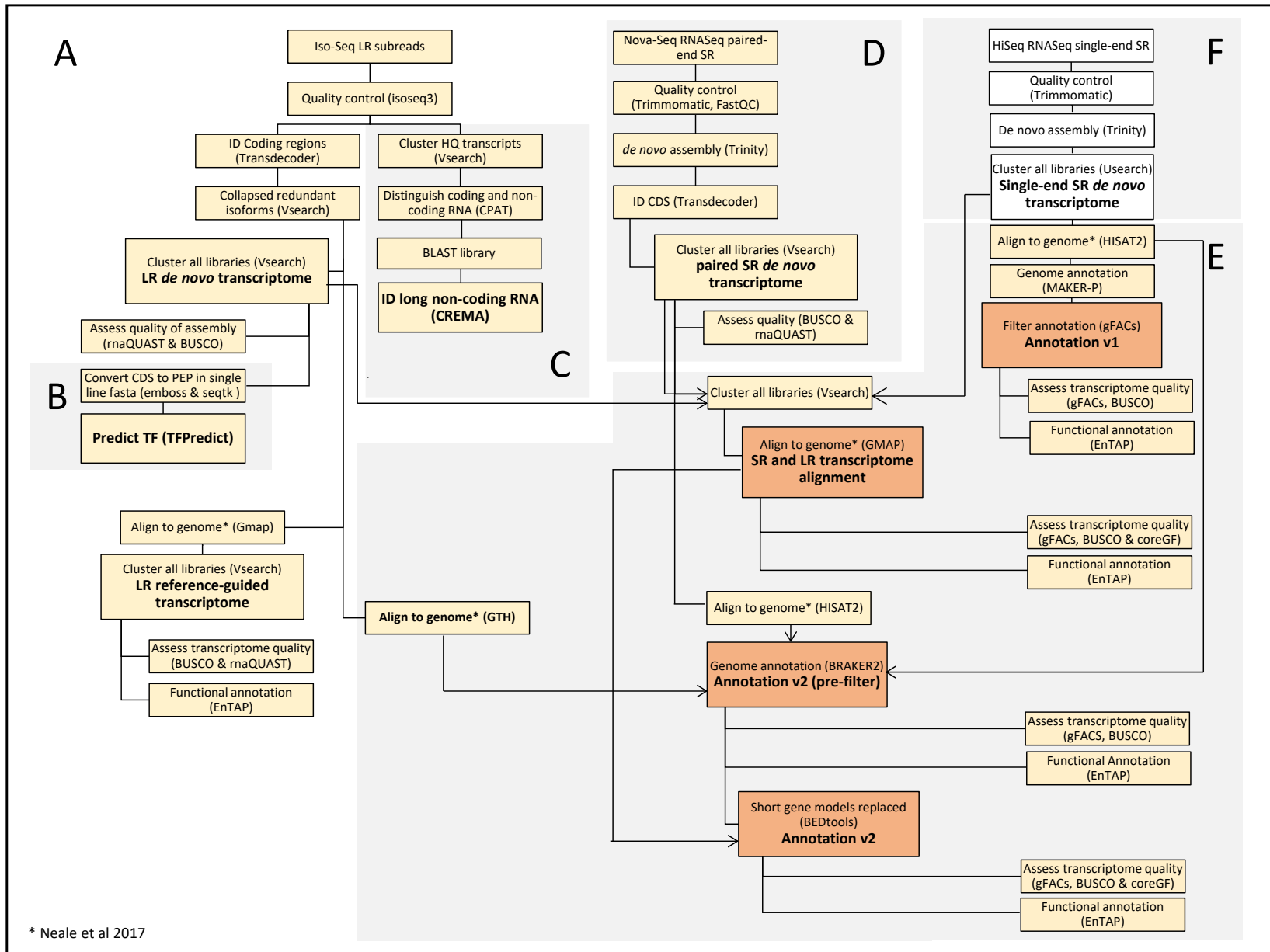

Supplement: jkac304_Supplementary_Data [file jkac304_supplementary_data.zip › Figure_S1_G3-2022-403847.pdf]
